# Extracellular matrix-mimetic ink for 3D printing and minimally invasive delivery of shape-memory constructs

**DOI:** 10.1016/j.mtbio.2026.102818

**Published:** 2026-01-16

**Authors:** Shima Tavakoli, Dimitra Pouloutidou, Oommen P. Oommen, Oommen P. Varghese

**Affiliations:** a Translational Chemical Biology Group, Science for Life Laboratory, Division of Macromolecular Chemistry, Department of Chemistry- Ångström Laboratory, Uppsala University, Uppsala SE75121, Sweden; b School of Pharmacy and Pharmaceutical Sciences, Cardiff University, CF10 3NB, United Kingdom

**Keywords:** Hyaluronic acid, Hydrogel, 3D printing, Shape-memory, Gallic acid

## Abstract

Direct injection of hydrogels loaded with therapeutics holds great promise for tissue regeneration; however, injectable hydrogels typically fill defect spaces without spatiotemporal control, which is critical for regenerating certain tissues. Conversely, 3D printing enables the fabrication of patterned hydrogel constructs but often requires invasive surgical implantation. Here, we present a novel strategy for the non-invasive delivery of 3D-printed constructs. Specifically, we developed gallic acid-modified hyaluronic acid (HA) that was crosslinked for the first time using potassium iodide (KI) as a catalyst, without the need for an initiator or light exposure. This also enabled protein conjugation with gelatin and collagen to obtain an extracellular matrix (ECM)-mimetic ink for 3D printing. We determined the distinct pKa values of the phenolic hydroxy groups of gallol-modified HA, which were utilized to achieve 3D printing at acidic pH, followed by efficient solution-free covalent crosslinking using ammonia gas to ensure complete crosslinking. This approach enabled efficient printing through fine nozzles (G32) and produced robust structures. The printed scaffolds were subsequently loaded into a larger needle and injected, demonstrating shape-memory properties by retaining their geometry post-injection. Furthermore, the scaffolds supported stem cell coating, where the stemness and differentiation of stem cells could be modulated by hydrogel composition and culture conditions, including chondrogenic differentiation towards cartilage-like constructs using TGF-β3. This strategy offers a versatile platform for developing HA-based hydrogels capable of protein conjugation, 3D printing, cell or biomolecule coating, and minimally invasive implantation while maintaining structural fidelity.

## Graphical abstract

**Figure:**
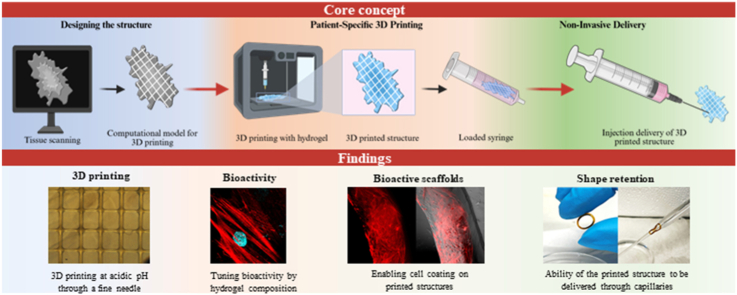

## 1 Introduction

3D printing offers a powerful prototyping technology that enables the fabrication of delicate and complex structures through a layer-by-layer deposition process. In the biomedical field, 3D printing integrates this manufacturing technique with materials and biomolecules to create functional tissue-like constructs [1,2]. Supported by advanced medical imaging and computer-aided design, bioprinters can precisely deposit “bioinks” to produce patient-specific implants with biomimetic architectures. Owing to its high geometric accuracy, customizability, and relatively simple operation, 3D bioprinting has gained wide application in engineering various tissues, including vasculature, cartilage, bone, nerve tissue, and skin [[1], [2], [3]]. Despite its transformative potential in tissue engineering and regenerative medicine, the clinical translation of 3D-printed constructs is often hindered by the need for invasive surgical implantation. In contrast, minimally invasive injection provides a promising alternative for delivering biomaterial implants directly to defect sites, reducing surgical risks such as infection, inflammation, and patient discomfort while also improving compliance [4,5]. However, conventional injectable systems face challenges in terms of low material retention, poor structural stability, and insufficient tissue integration [6]. Moreover, for the regeneration of complex tissues, such as bone and cartilage, the shape and geometry of the implant play a critical role in guiding functional tissue repair [7,8].

Hydrogels, owing to their high water content and tissue-like mechanical properties, have emerged as promising candidates for minimally invasive delivery of therapeutics by simple injection [9]. Their biomimetic nature allows them to function as living tissue analogs, supporting cell encapsulation and tissue regeneration. However, in situ polymerization in vivo often presents challenges in precisely controlling the location of gelation, gelation kinetics, mechanical strength, geometry, and microstructure [5]. Although traditional hydrogels can be prefabricated in vitro using 3D printing technologies, their clinical application typically requires invasive surgical implantation. Furthermore, these printed hydrogels are often bulky and dense, making them prone to clogging during delivery by injection or becoming damaged upon delivery, compromising their function [10,11]. In our previous studies, we demonstrated that in vivo injection of recombinant human bone morphogenetic protein-2 (rhBMP-2) loaded bulk hydrogel in a subcutaneous or subperiosteal model supported tissue growth; however, such soft gels lack shape retention in newly formed bone tissue, highlighting the importance of geometric fidelity in achieving effective regeneration outcomes [[12], [13], [14]]. To make 3D-printed hydrogel constructs suitable for injection-based delivery and tissue repair, they must be capable of adapting to defect geometries while retaining their designed structure and functionality. Thus, delivering 3D-printed structures to defect sites through a non-invasive approach while ensuring both excellent injectability and shape-recovery after injection remains an unmet challenge. Few attempts have been made to deliver 3D-printed structures non-invasively using shape-memory hydrogels [15]. While there have been successful reports on the development of shape-memory hydrogels, their shape recovery typically requires an external trigger, such as the presence of fluidics, temperature change, light radiation, and pH variation, which restricts their applicability in vivo [15,16]. Additionally, freeze-drying of these gels, often used to enhance their shape-memory properties, can negatively impact their geometry and microstructure [17].

To develop an ideal ink for 3D printing, the hydrogel must possess sufficient initial viscosity to maintain the printed structure but not be too viscous to clog the needle and disrupt the printing process [18,19]. In addition, for a hydrogel to exhibit shape-memory properties, it must have strong mechanical integrity with robust crosslinks and high porosity to prevent bond breakage or structural failure [20]. Hyaluronic acid (HA), a major component of the extracellular matrix (ECM), is widely utilized in the design of hydrogels for 3D printing due to its excellent biocompatibility and versatility [1]. HA-based hydrogels are often engineered using various crosslinking methods, including dynamic covalent chemistry approaches, which are particularly attractive for 3D bioprinting. This is largely due to the shear-thinning and self-healing properties of dynamic bonds, that is essential for successful 3D printing [21]. We have recently demonstrated that disulfide-linked HA hydrogels could be used for bioprinting human stem cells and creating in vitro osteoarthritis models [19]. However, these hydrogels are soft and do not exhibit shape-memory properties as the dynamic bonds can break and reform, altering the final geometry of the printed structure. While dynamic crosslinking is favored for 3D printing due to its ability to facilitate printability, stronger non-dynamic covalent bonds are essential for creating a shape-memory hydrogel that can withstand shear forces during injection and retain its geometry.

**Fig. 1:**
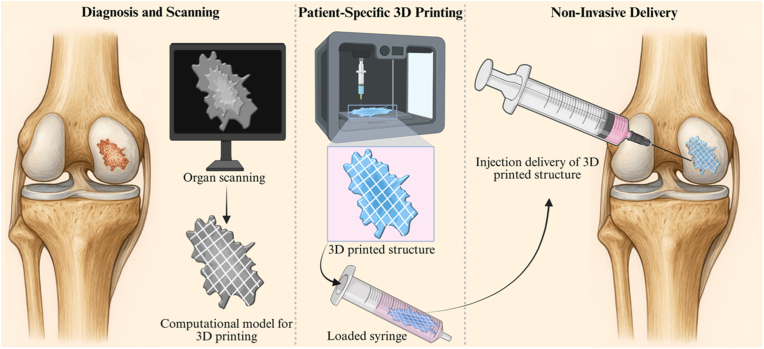
Representative illustration demonstrating potential integration of 3D printing and non-invasive delivery of a 3D printed scaffold via injection. First, the injury site is imaged and digitally reconstructed to generate a patient-specific 3D model. Using an optimized shape-memory hydrogel, a porous, anatomically tailored implant is printed. Following crosslinking, the structure is compressed and loaded into a syringe, enabling minimally invasive injection into the target site, where it recovers its original shape and architecture.

In this study, we present a novel approach for engineering injectable, 3D-printable hydrogels by chemically modifying HA with gallic acid (GA) derivative, namely gallic hydrazide conjugates with HA carboxylates. GA, a naturally occurring polyphenolic compound with three hydroxyl groups on its aromatic ring, is well known for its antioxidant capacity and tissue-adhesive properties [22,23]. Under aqueous conditions, GA can scavenge free radicals and undergo oxidative crosslinking to form robust hydrogel networks. We have shown previously that this crosslinking proceeds through radical formation and quinone intermediates, governed by the differential pKa of GA hydroxyls [22]. Building on this, we developed a strategy to 3D print GA-grafted HA hydrogels at mildly acidic pH, where initial radical generation and quinone formation induce partial crosslinking, imparting sufficient viscosity for shape fidelity during printing without full gelation. Post-printing, crosslinking was completed through exposure to ammonia gas, which raises the pH and triggers further oxidation of GA without exposure to any solvents. Potassium iodide (KI) was incorporated into the formulation to initiate radical generation and promote the formation of an efficient quinone intermediate. The resulting printed hydrogels demonstrated excellent long-term stability and mechanical integrity. Furthermore, the system allows facile incorporation of bioactive proteins via GA conjugation, and the printed constructs can be loaded into syringes and delivered to target sites while retaining their defined geometry and internal microarchitecture (Fig. 1). This approach has potential for minimally invasive delivery of 3D-printed scaffolds, particularly in applications such as cartilage repair, where preservation of scaffold geometry is essential for guiding organized tissue regeneration.

## 2 Results and discussion

### 2.1 Synthesis and characterization of HA modification with GA

To design a shape-memory ECM-based ink for 3D printing, we envisioned that the pH-driven phenolic radical formation of GA, grafted onto the HA backbone, yields a hydrogel with suitable initial viscosity for printing at low pH while ensuring high structural integrity post-printing at higher pH. For this purpose, gallic hydrazide was synthesized following the previously reported protocol [23], which was conjugated to the carboxylate group of β-1,4 glucuronic acid in the HA disaccharide unit. For the synthesis of HA-GA, we employed carbodiimide coupling chemistry, as illustrated in the Supporting Information (Fig. S1). Briefly, the carboxyl groups of HA were activated using water-soluble 1-ethyl-3-(3-dimethylaminopropyl)-carbodiimide hydrochloride (EDC) with 1-hydroxybenzotriazole hydrate (HOBt) as a catalyst that yielded an active ester, facilitating the nucleophilic substitution with gallic hydrazide to obtain the desired HA-GA product. The grafting of GA onto the HA backbone was assessed spectroscopically using both ^1^H NMR and UV–Vis. The degree of modification was quantified by the ratio of the relative peak areas of the aromatic proton peak of GA at 6.89 ppm to the methyl proton peak of the *N*-acetyl group of HA at 1.95 ppm (Supporting Information, Fig. S2), which was determined to be ∼20 % with respect to the disaccharide repeating units. This level of substitution was considered optimal, as a lower degree of modification results in weak hydrogel formation due to insufficient crosslinking. Conversely, a higher degree of modification could lead to overly rapid reaction kinetics, which may hinder effective mixing and prevent proper crosslinking between reactive groups [18,24]. Moreover, excessive modification might compromise the native biological properties of HA, affecting its biocompatibility and cellular interactions [25,26]. UV–Vis analysis also exhibited a broad absorbance at 325 nm (Supporting Information, Fig. S3), corresponding to the deprotonation of the phenolic proton of GA at the para position, further confirming the successful conjugation of GA on the HA backbone [22].

The pH dependence of GA has been previously reported in the literature [27,28]. It has been demonstrated that the initial pKa value of GA corresponds to the deprotonation of the carboxyl group, which in our case has been substituted by hydrazide groups for conjugation to HA. The computational studies indicate that the deprotonation involves the phenolic hydroxyl group at the para position (first pKa for GA hydrazide), which exhibits a lower Gibbs free energy compared to the phenolic hydroxyl group at the meta position [27]. The other pKa value (second pKa for GA hydrazide) corresponds to the deprotonation of the two symmetrical phenolic hydroxyl groups located at the meta positions of the aromatic ring [22,28,29]. In one study, Hostnik et al. showed through simulations that different populations of ionized species of GA coexist at various pH values, with their results aligning well with experimentally obtained spectra [28]. They demonstrated that at pH 2.04, GA exists in its protonated form. As the pH increases to 6.01, the singly deprotonated species becomes predominant, corresponding to the pKa of para-hydroxyl groups of GA. At pH 8.98, which is above the meta-hydroxyl pKa, the doubly deprotonated ionic species dominate, while some singly deprotonated and triply deprotonated species also coexist. Notably, at these higher pH values, GA is prone to oxidation.

**Fig. 2:**
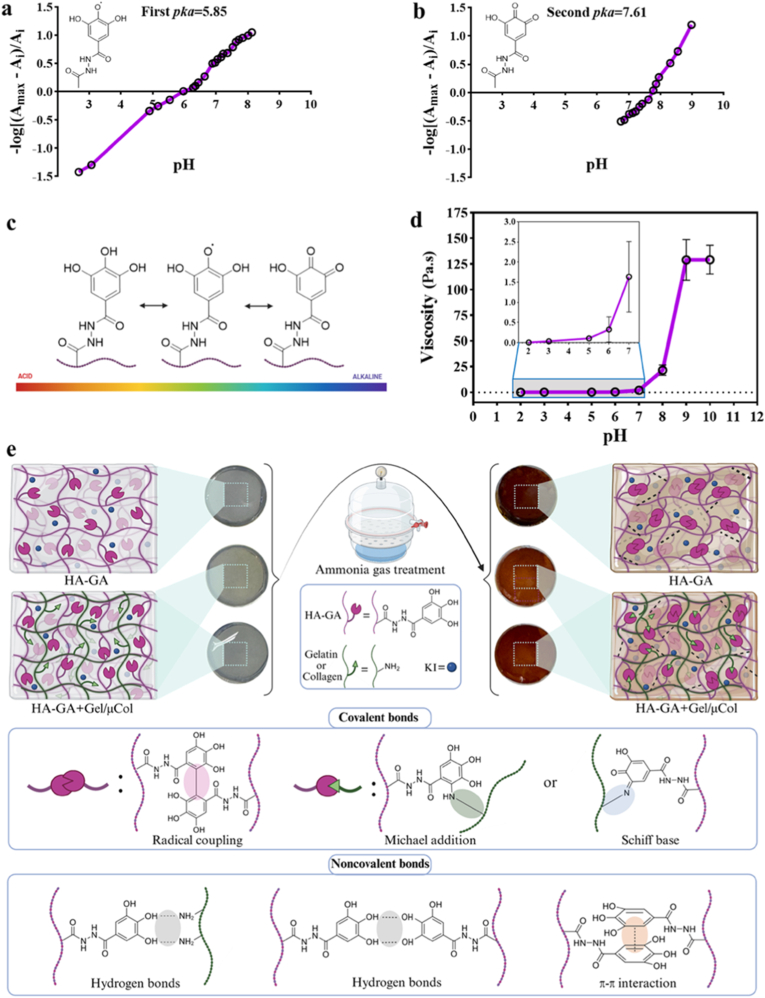
Chemical characterization and hydrogel formation. (a, b) UV–Vis analysis used to determine the first and second p*K*a values of gallic acid (GA) derivatives conjugated to hyaluronic acid (HA), corresponding to phenolic deprotonation and oxidation-induced crosslinking, respectively. (c) Structural depiction of GA-conjugated HA (HA-GA) at a range of pH and the oxidation-induced formation of reactive quinone intermediates upon pH elevation. (d) Viscosity of HA-GA hydrogel precursor at a range of pH from 2 to 10, the inset represents the zoom of the pH range of 2–7. (e) Schematic illustration of the crosslinking process in a sealed ammonia gas chamber, showing the transition of the hydrogel from transparent to brown. The panel also highlights key covalent and noncovalent interactions in the hydrogel network, facilitated by potassium iodide (KI), which promotes radical formation and crosslinking. (For interpretation of the references to color in this figure legend, the reader is referred to the Web version of this article.)

In this study, we experimentally determined the pKa of the GA derivative when conjugated to HA. UV–Vis absorbance spectra of HA-GA solutions were recorded across a range of pH values, as shown in Supporting Information, Fig. S4. The spectra exhibited a broad peak at 325 nm, which became more pronounced at pH values above 5. This shift corresponds to the first pKa, attributed to the deprotonation of the phenolic hydroxyl group located at the para position of the aromatic ring in the GA moiety, with a calculated pKa value of 5.85 (Fig. 2a). At more basic pH, an additional increase in absorbance was observed around 420 nm, corresponding to the oxidation of GA groups conjugated to the HA backbone. This oxidation promotes radical formation and subsequent intermolecular dimerization. Based on these observations, the second pKa was calculated to be 7.61 (Fig. 2b). These pKa values are well-suited for our 3D printing strategy. The first pKa allows for partial oxidation, leading to a moderate viscosity at mildly acidic pH (around 5.5–6), enabling smooth extrusion and temporary shape retention of the printed structure without needle clogging (Fig. 2c). Subsequent elevation of the pH completes the oxidation and crosslinking process, resulting in a mechanically stable hydrogel. This two-stage crosslinking mechanism is also supported by our viscosity measurements (Fig. 2d), which show a moderate increase in viscosity near the first pKa and a more substantial rise around the second pKa, as shown in the inset with higher magnification, aligning with the expected crosslinking behavior.

To obtain fully crosslinked hydrogel structures after printing, the pH was elevated above the second pKa of the GA derivative using an ammonia gas-based method, where aqueous ammonia was allowed to equilibrate with its vapor phase in a sealed chamber. This novel solution-free method was envisioned because, while the printed filaments could temporarily retain their shape, completing the oxidative crosslinking at a higher pH was necessary to ensure long-term structural fidelity without any possibility of swelling. Moreover, adjusting the pH through the addition of alkaline solution was not efficient, as it led to rapid reaction kinetics resulting in inhomogeneous mixing within the viscous hydrogel precursor, resulting in heterogeneous and irreproducible gel formation. Since GA could also facilitate site-specific conjugation with other nucleophilic groups, we envisioned designing 3D printed matrices having different protein-based components. Specifically, we developed hydrogel-based bioinks that included HA modified with GA derivatives (HA-GA), HA-GA combined with gelatin (HA-GA.Gel), and HA-GA combined with collagen microfibers (HA-GA.μCol). To promote efficient crosslinking and bioconjugation, we supplemented all precursor solutions with 50 mM KI, which served as both a radical initiator and oxidizing agent. We have recently shown that 50 mM KI was ideal to facilitate disulfide formation in a concentration-dependent manner [19], and this prompted us to evaluate KI as a catalyst for gallol chemistry, as this is also a radical-based strategy. Interestingly, we observed that KI also contributed to enhanced dimerization of GA moieties on the HA backbone, thereby increasing crosslinking density. While previous studies have demonstrated that HA-GA can be crosslinked using blue light in the presence of riboflavin as a photo-initiator, enabling conjugation to proteins such as collagen, our approach utilizes just KI to achieve similar outcomes [30]. The KI-mediated radical chemistry also promoted protein conjugation to GA without the need for light exposure or additional initiators, offering a versatile strategy for grafting a variety of proteins onto HA. Such hydrogel formation process allows for efficient mixing of the hydrogel precursors and KI, as both are in liquid form. This enables uniform hydrogel formation after ammonia gas treatment, as indicated by the color change of the hydrogels shown in Fig. 2e. In all hydrogels, we expect the dimerization of the GA group through a quinone intermediate [22,31]. For the HA-GA.Gel and HA-GA.μCol hydrogels, a Michael addition and Schiff base reactions are also plausible. These reactions happen between the amino groups of gelatin and collagen and the reactive quinone intermediate at the ortho and meta/para positions of the GA group. Additionally, the formation of noncovalent interactions is also expected in these hydrogel systems [32]. These include hydrogen bonding between the phenolic hydroxyls of the GA groups in HA-GA across all hydrogels, as well as between the phenolic hydroxyls of HA-GA and the amino groups of gelatin and collagen in HA-GA.Gel and HA-GA.μCol hydrogels, respectively. Furthermore, aromatic interactions due to the stacking of the GA groups in HA-GA are also expected. All together, these interactions lead to the formation of a strong crosslinked hydrogel network following ammonia gas treatment. Such a gas treatment is expected to yield highly crosslinked hydrogels with a basic pH, which is not conducive to biological applications. To address this, the hydrogels were thoroughly washed and allowed to rest in PBS for 24 h. This process effectively removed excess ammonia and gradually brought the pH down to a physiologically relevant level suitable for subsequent cell culture applications.

### 2.2 Rheological characterization of hydrogels

**Fig. 3:**
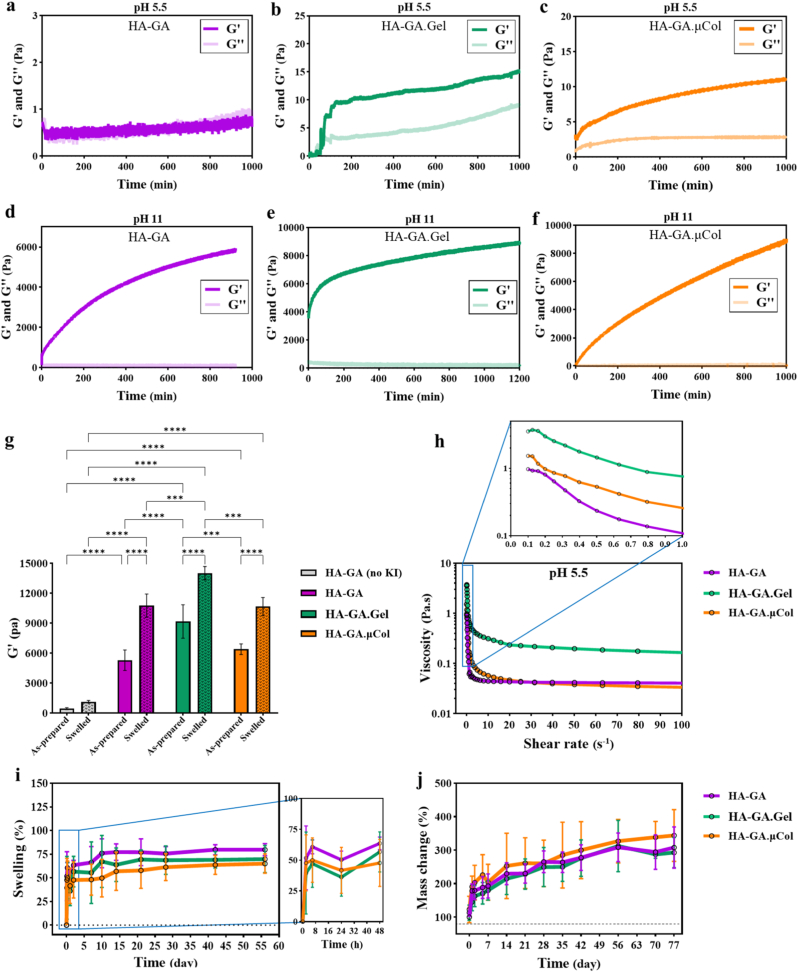
Rheological and physical characterization of hydrogels. (a–c) Time sweep rheological analysis of hyaluronic acid conjugated with gallic acid derivative (HA-GA), HA-GA with gelatin (HA-GA.Gel), and HA-GA with collagen microfibers (HA-GA.μCol) at acidic pH 5.5 in the presence of KI. (d–f) Time sweep measurements of the same hydrogel formulations at basic pH 11, demonstrating enhanced crosslinking kinetics under alkaline conditions. (g) Storage modulus (G′) of fully crosslinked hydrogels measured immediately after ammonia gas treatment (as-prepared) and after 24 h equilibration in PBS (swelled). Statistical analysis is obtained from a two-way ANOVA analysis, significance is represented as: ∗ = p < 0.05, ∗∗ = p < 0.01, ∗∗∗ = p < 0.001, ∗∗∗∗ = p < 0.0001. (h) Shear-thinning behavior of hydrogel precursors at pH 5.5; the inset highlights initial viscosity. (i) Swelling ratio of crosslinked hydrogels in PBS at 37 °C over 56 days. (j) Enzymatic degradation profile and long-term stability of hydrogels in the presence of hyaluronidase at 37 °C over 77 days.

To investigate the role of pH in catalyzing the oxidation of GA groups and influencing gelation kinetics, time sweep measurements were performed using a rheometer at two pH conditions, namely at pH 5.5 and pH 11. The gelation point was determined by identifying the crossover of the storage modulus (G′) and the loss modulus (G″), indicating the transition from a liquid-like to a solid-like behavior. As shown in Fig. 3a–c, gelation kinetics at pH 5.5 were relatively slow across all hydrogel compositions. Surprisingly, in formulations containing gelatin or μCol, we observed an instantaneous gelation kinetics that began almost immediately after mixing, even under acidic conditions. Despite the rapid start, these gels remained weakly crosslinked, as reflected by their low final storage modulus. In contrast, at basic pH (pH 11), all hydrogel formulations exhibited rapid gelation, achieving their maximum stiffness by the end of the time sweep (Fig. 3d–f). These results suggest that while gelatin and μCol can induce early-stage physical interactions or partial crosslinking even under acidic conditions, alkaline environments significantly accelerate oxidative crosslinking of GA groups in all hydrogel systems.

To further examine the effects of KI and different hydrogel compositions on mechanical properties, we performed amplitude sweep tests to measure the G′ of fully crosslinked hydrogels, both immediately after crosslinking (as-prepared) and following 24 h of washing/swelling in PBS (swelled). As shown in Fig. 3g, HA-GA hydrogels without KI exhibited a relatively low G′ of 459 ± 101 Pa, which increased to 1143 ± 126 Pa after swelling. Upon the addition of KI, the stiffness of HA-GA hydrogels increased significantly, reaching 5305 ± 1025 Pa and 11085 ± 983 Pa for as-prepared and swelled samples, respectively. This enhancement was also evident in the appearance of the hydrogels, where samples without KI exhibited a yellow color, indicating incomplete oxidation of GA, while those with KI turned brown, reflecting more effective oxidation of the GA groups leading to dimerization (Supporting Information, Fig. S5). This demonstrates that KI is essential for generating sufficient radicals to promote effective oxidative coupling of GA moieties. Incorporating gelatin into the HA-GA system with KI further enhanced stiffness, with the storage modulus exceeding ∼14000 Pa after swelling. This significant increase is likely due to additional covalent crosslinking between the amino groups of gelatin and the gallol moiety on HA, which is facilitated by the presence of KI. However, the increase in stiffness was less pronounced in the μCol group (∼11000 Pa). This may be attributed to the smaller fragments of μCol and its lower concentration relative to gelatin, which limits the extent of additional crosslinking. Similarly, we demonstrated that reducing the solid content of HA-GA from 4 % to 2 % resulted in a significant decrease (almost two times decrease) in the final storage modulus (Supporting Information, Fig. S6), indicating that the stiffness of the hydrogels can be effectively tuned by adjusting the concentration. Interestingly, in all groups, the storage modulus increased substantially after 24 h of swelling as the polymeric chains underwent extension, which increases the elasticity of the network and, consequently, the stiffness [33]. This increase in storage modulus results in a stiffer and denser network structure, thereby reducing the ability of water molecules to further penetrate the hydrogel matrix. Moreover, to better mimic in vivo relevant conditions, the hydrogel samples were incubated in complete cell culture medium at 37 °C before the mechanical evaluation was performed at 37 °C. The structural integrity of all hydrogels remained intact after incubation (Supporting Information, Fig. S7a). Interestingly, their stiffness increased compared to samples incubated in PBS. The measured elastic moduli reached 12294 ± 2516 Pa, 16368 ± 1668 Pa, and 14027 ± 768 Pa for HA–GA, HA–GA.Gel, and HA–GA.μCol, respectively (Supporting Information, Fig. S7b). These results indicate that exposure to biologically relevant media does not compromise the mechanical stability of the hydrogels. Instead, it further reinforces the network, likely due to additional physical interactions between medium proteins and the HA/GA moieties.

Next, the effect of hydrogel composition on the initial viscosity and shear-thinning behavior was evaluated at an acidic pH of 5.5, close to the first pKa of the GA moiety, which is also the pH used during 3D printing. Shear-thinning behavior, where the viscosity of material decreases with increasing shear rate, is a desirable property for injectable hydrogels and 3D printing applications, as it enables smooth extrusion through fine nozzles while maintaining shape fidelity post-deposition [34]. As shown in Fig. 3h (inset), the initial viscosities of the hydrogel formulations varied depending on composition. At a shear rate of 0.1 s^−1^, the HA-GA hydrogel containing KI exhibited a viscosity of 0.97 Pa s. The addition of gelatin and μCol increased the initial viscosity to 3.5 Pa s and 1.5 Pa s, respectively. This suggests that the addition of gelatin or μCol could not only increase the net solid content but also presumably interact with HA-GA via hydrogen bonding or electrostatic interactions, making it suitable for developing bioink. Upon increasing the shear rate from 0.1 to 100 s^−1^, all hydrogel formulations displayed a noticeable reduction in viscosity (Fig. 3h), confirming their shear-thinning behavior. This rheological property is particularly advantageous for extrusion-based 3D printing, especially when using fine-gauge needles such as 32G (inner diameter of 108 μm), as it ensures ease of extrusion without clogging while allowing the printed constructs to maintain their shape post-printing [19].

### 2.3 Swelling and long-term stability of hydrogels

We determined the swelling percentage of HA-GA, HA-GA.Gel and HA-GA.μCol at physiological conditions for over 56 days. Fig. 3i indicates that all hydrogels showed similar swelling profiles. Particularly, all hydrogels swelled at a swelling capacity of ∼50 % in the first 8 h, and reached a steady state after 24 h of swelling study. Subsequently, all hydrogels demonstrated remarkable stability for the following 56-day study period. During this period, the HA-GA.Gel and HA-GA.μCol hydrogels remained stable at 69.9 ± 14.3 % and 65.1 ± 10.1 % swelling, respectively, while HA-GA exhibited a slight increase to 79.8 ± 6.3 %. However, all hydrogels maintained their shape without any visual signs of swelling. This observation can be attributed to the fact that, following a 24 h swelling, the storage modulus of HA-GA, HA-GA.Gel, and HA-GA.μCol increased, and that correlates with the swelling degree by the synergistic effect of the enhanced elasticity of the network, combined with the formation of new crosslinks between GA groups that become accessible for interaction. Additionally, the stability of the hydrogels was evaluated under acidic conditions (pH = 4). As shown in Supporting Information, Fig. S8, all formulations maintained their structural integrity without noticeable deformation in the acidic environment. These results further demonstrate the robustness of the hydrogel network across a wide pH range, supporting its potential applicability in diverse biomedical settings.

Since HA-based materials are known to be degraded by a ubiquitous enzyme, namely hyaluronidase, we assessed the enzymatic stability of the hydrogels in hyaluronidase solution. To our surprise, none of the hydrogels underwent degradation for over 77 days under our experimental conditions, although we observed some swelling (Fig. 3j). Although hyaluronidase is known to degrade gels, in our case, the presence of a dense crosslinked network restricted the diffusion of the enzyme into the hydrogel, similar to our previous observation with carbohydrazide crosslinked hydrogels [35,36]. Interestingly, the degradation properties of the hydrogels could be reversed by simply decreasing the solid content of the hydrogels. Lowering the solid content reduced the crosslinking density between chains, which allowed hyaluronidase molecules to penetrate the hydrogel matrix and degrade the hydrogel in a shorter period (Supporting Information, Fig. S9). This behavior indicates that the swelling and degradation profile of these hydrogels is controllable and can be tuned by adjusting the solid content, which results in altering the crosslinking density. Also, degradation behavior is directly correlated with network density and enzyme accessibility, as a denser network can restrict the enzyme penetration and result in a slower/limited degradation.

### 2.4 Effect of hydrogel matrix on human stem cells

**Fig. 4:**
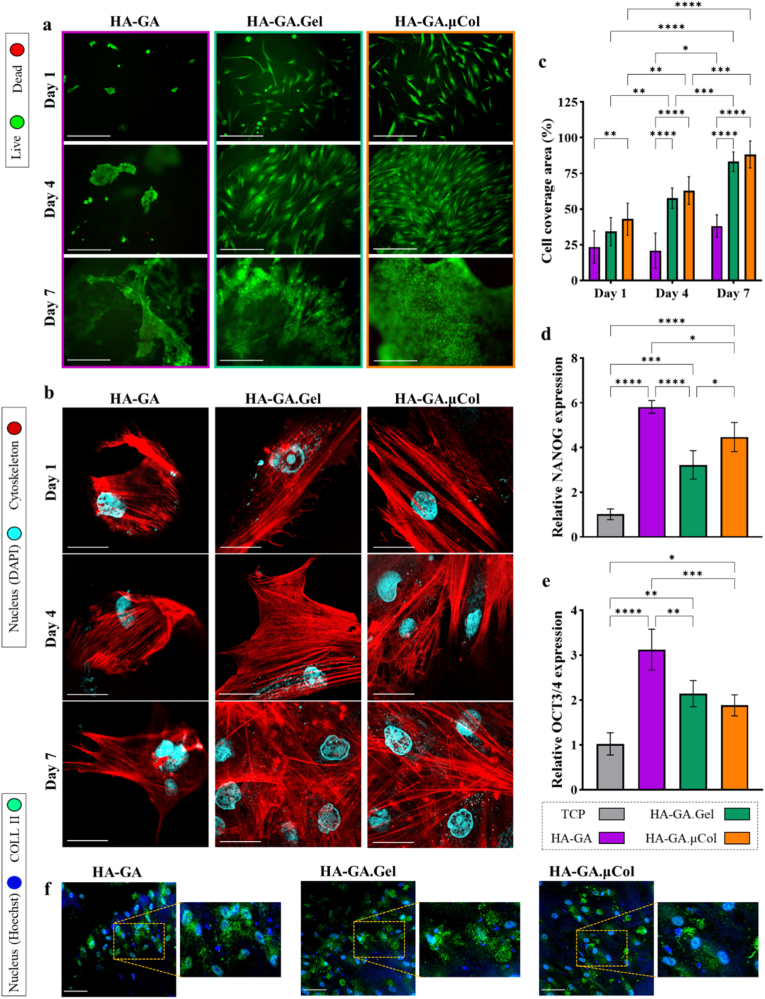
Hydrogel–cell interactions. (a) Live/Dead staining of human mesenchymal stem cells (hMSCs) seeded on HA conjugated with gallic acid (HA-GA), HA-GA with gelatin (HA-GA.Gel), and HA-GA with collagen microfibers (HA-GA.μCol) over 1, 4, and 7 days (scale bar: 150 μm). (b) Cytoskeletal (phalloidin) and nuclear (DAPI) staining of hMSCs on each hydrogel at the same time points, illustrating cell morphology and spreading (scale bar: 25 μm). (c) Quantification of hydrogel surface area covered by cells over time, calculated using ImageJ. Statistical analysis is obtained from a two-way ANOVA analysis, significance is represented as: ∗ = p < 0.05, ∗∗ = p < 0.01, ∗∗∗ = p < 0.001, ∗∗∗∗ = p < 0.0001. (d–e) Relative gene expression of stemness markers NANOG and OCT3/4 in hMSCs after 4 days of culture on different hydrogel formulations. Statistical analysis is obtained from an ordinary one-way ANOVA analysis, significance is represented as: ∗ = p < 0.05, ∗∗ = p < 0.01, ∗∗∗ = p < 0.001, ∗∗∗∗ = p < 0.0001. (f) Collagen II (COLL II) immunostaining of hMSCs on each hydrogel after 14 days in chondrogenic media (scale bar: 60 μm).

Biocompatibility of the developed hydrogel matrices with human stem cells is a critical factor for their application in regenerative medicine [37]. To assess this, human mesenchymal stromal cells (hMSCs) were seeded onto preformed HA-GA, HA-GA.Gel, and HA-GA.μCol hydrogels in a 48-well plate. Cell viability and morphology were evaluated on days 1, 4, and 7. At each time point, the hydrogels were gently washed with PBS to remove debris, followed by staining with a Live/Dead assay and imaging under a fluorescence microscope. As illustrated in Fig. 4a, viable cells are indicated by green fluorescence, while the dead cells appear red. Across all samples and time points, the majority of cells remained viable, demonstrating the high cytocompatibility of the hydrogel systems. Notably, at day 1, hMSCs seeded on HA-GA.Gel and HA-GA.μCol exhibited early attachment, elongation, and spreading across the matrix surface. In contrast, cells seeded on HA-GA alone showed minimal attachment and instead formed small, rounded aggregates. This difference in cell behavior is attributed to the presence of bioactive adhesion sites in gelatin and μCol, such as RGD motifs, which facilitate integrin-mediated cell attachment and spreading [38]. While HA can interact with cells via CD44 receptors, this alone is not sufficient to promote effective cell spreading, leading to the observed cell clustering on HA-GA hydrogels [39]. By days 4 and 7, the aggregates on HA-GA became more prominent as additional cells clustered together. Meanwhile, on HA-GA.Gel and HA-GA.μCol, hMSCs continued to proliferate and spread uniformly across the surface with the formation of continuous cellular films across the hydrogel surface. For longer time points, cytotoxicity arising from degradation is not expected, as the hydrogel formulations are composed solely of HA, gelatin, or collagen, which are well-established biocompatible and biodegradable biomaterials. Their degradation products, such as small peptides and hyaluronan oligosaccharides, are generally non-toxic and are naturally metabolized in vivo.

To further investigate the cytoskeletal organization and morphological characteristics of hMSCs on different hydrogel matrices, cells were stained with phalloidin (to visualize F-actin filaments) and DAPI (to label cell nuclei) at days 1, 4, and 7. Confocal microscopy imaging (Fig. 4b) revealed distinct differences in cell morphology across the different hydrogel compositions. In the HA-GA group, cells largely retained a rounded morphology with limited cytoskeletal organization, indicating minimal adhesion and spreading on the matrix. By day 7, more cells had clustered into larger aggregates, and some degree of cell elongation was observed within these clusters, suggesting delayed or limited cell-matrix interaction over time. In contrast, hMSCs seeded on HA-GA.Gel and HA-GA.μCol hydrogels displayed significantly enhanced spreading, characterized by well-developed F-actin networks and an elongated morphology as early as day 1. The F-actin staining showed broad, organized filaments, indicative of strong cytoskeletal tension and robust cell adhesion. By day 7, both matrices were densely covered with proliferated cells, and some multicellular aggregates formed, likely a result of the limited available surface area due to the high cell density. Additionally, to quantitatively assess cell spreading and coverage, we analyzed the percentage of hydrogel surface area occupied by cells over time using ImageJ. As shown in Fig. 4c, the HA-GA hydrogel exhibited the lowest surface coverage, with only minimal attachment and almost no spreading of cells throughout the culture period. However, by day 7, cell-covered areas increased to approximately 38 %, likely due to gradual aggregation and growth within clusters. In contrast, both HA-GA.Gel and HA-GA.μCol groups showed a progressive and significant increase in surface coverage. At day 7, 83 % and 88 % of the hydrogel surface was covered by cells in HA-GA.Gel and HA-GA.μCol, respectively. These results reinforce the important role of proteins like gelatin and collagen in promoting cell adhesion, spreading, and proliferation through enhanced cell–matrix interactions.

Next, to investigate the effect of matrix composition on the stemness of seeded hMSCs, as well as the impact of cell adhesion versus aggregation on stem cell reprogramming, we analyzed the expression of the key pluripotency markers NANOG and OCT3/4 after 4 days of culture using quantitative PCR (qPCR). These transcription factors are essential for maintaining the self-renewal and undifferentiated state of stem cells and are widely expressed across different stem cell types, including hMSCs [40]. As shown in Fig. 4d and e, when compared to cells cultured on tissue culture plates (TCP), the expression levels of both stemness markers significantly increased across all hydrogel groups. This enhancement is likely due to the lower mechanical stiffness of the hydrogels relative to TCP, which creates a more favorable microenvironment for maintaining stemness. Additionally, the interaction between HA and the CD44 receptor on hMSCs has been previously reported to activate the Rho/ROCK signaling pathway, known to support self-renewal and stem-like characteristics [41,42]. Interestingly, among all hydrogel compositions, HA-GA alone resulted in the most significant upregulation of both NANOG (5.8 folds increase) and OCT3/4 (3.1 folds increase), suggesting that this matrix may provide the most conducive environment for stemness preservation. In the HA-GA.Gel and HA-GA.μCol groups, expression of NANOG increased by 3.2- and 4.5-fold, while OCT3/4 increased by 2.1- and 1.9-fold, respectively. Although these values are higher compared to cells on TCP, stemness was best preserved on HA-GA alone. This highlights that integrin-mediated cell attachment in protein-containing hydrogels promotes cell spreading, which in turn can affect stemness maintenance [38]. This finding is consistent with previous studies showing that HA-based hydrogels can enhance stemness marker expression compared to 2D culture systems, largely due to their ability to promote cellular aggregation rather than spreading [43,44]. It is shown that cell shape and cytoskeletal tension play a key role in stem cell fate; elongated cells with prominent actin stress fibers tend to differentiate, while rounded cells with minimal tension are more likely to retain stem-like properties [[45], [46], [47]]. The limited cell spreading and increased aggregation observed in HA-GA likely reduce YAP/TAZ nuclear translocation, further supporting a stem-like state in hMSCs.

While all tested hydrogels preserved hMSC stemness more effectively than TCP, the presence of specific biological cues in the surrounding environment can direct these cells toward targeted differentiation. In this study, we demonstrated that when hMSCs were seeded on the hydrogels and cultured in chondrogenic medium containing TGF-β3, they produced substantial amounts of collagen II after 14 days, indicating successful chondrogenesis. As shown in Fig. 4f, collagen II was detected in all groups, including HA-GA, HA-GA.Gel, and HA-GA.μCol. However, hydrogels composed solely of HA generally retained fewer cells on their surface, consistent with earlier observations. The detection of collagen II across all formulations confirms that these hydrogels, regardless of composition, can be coated with cells and, under appropriate culture conditions, support differentiation toward targeted tissue types. Also, the quantitative measurement of normalized fluorescent intensity showed no significant difference among the compositions (Supporting Information, Fig. S10). This capability highlights their potential to generate in vitro pre-matured tissue models, such as cartilage in this case, which could subsequently be delivered non-invasively for therapeutic applications. Of note, ex vivo cultured tissue models are currently being evaluated in a phase 2 clinical trial for treating cartilage defects in OA patients [48].

### 2.5 Effect of hydrogel on radical scavenging and intracellular ROS

**Fig. 5:**
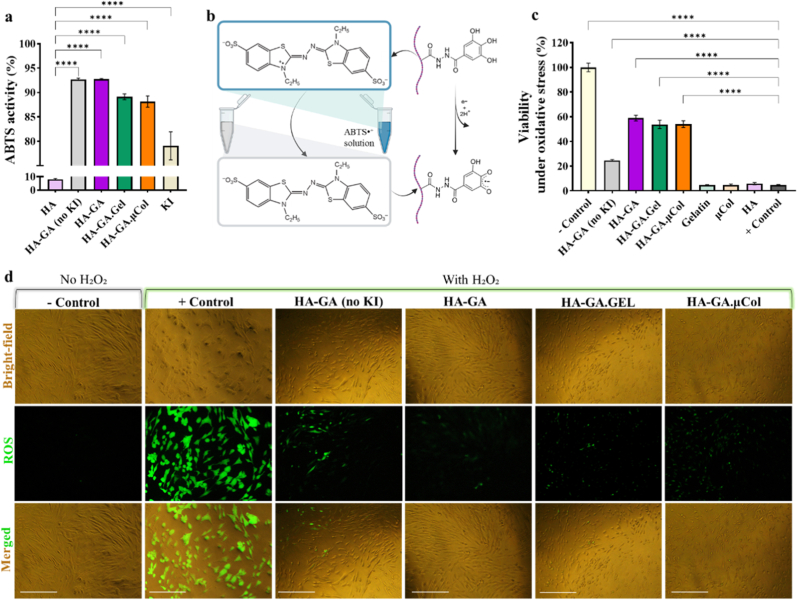
Antioxidant properties and reactive oxygen species (ROS) scavenging capacity of hydrogel formulations. (a) Quantification of ABTS•^+^ radical scavenging efficiency for HA conjugated with gallic acid (GA) derivative (HA-GA), HA-GA with gelatin (HA-GA.Gel), and HA-GA with collagen microfibers (HA-GA.μCol), in the presence of potassium iodide (KI). Statistical analysis is obtained from an ordinary one-way ANOVA analysis, significance is represented as: ∗ = p < 0.05, ∗∗ = p < 0.01, ∗∗∗ = p < 0.001, ∗∗∗∗ = p < 0.0001. (b) Schematic illustration of the electron transfer reaction between GA moieties in HA-GA and the ABTS•^+^ radical cation. (c) Cell viability under oxidative stress conditions, assessed using the PrestoBlue assay, after treatment with different hydrogel compositions. Statistical analysis is obtained from an ordinary one-way ANOVA analysis, significance is represented as: ∗ = p < 0.05, ∗∗ = p < 0.01, ∗∗∗ = p < 0.001, ∗∗∗∗ = p < 0.0001. (d) Fluorescence microscopy images showing intracellular ROS levels in cells treated with hydrogel formulations under oxidative stress, where green fluorescence indicates ROS presence (scale bar: 150 μm). (For interpretation of the references to color in this figure legend, the reader is referred to the Web version of this article.)

To evaluate the antioxidant and radical scavenging properties of the hydrogel compositions, we employed the ABTS (2,2′-azino-bis(3-ethylbenzothiazoline-6-sulfonic acid)) radical scavenging assay. This electron transfer-based method quantifies total antioxidant capacity by measuring the ability of compounds to reduce the dark blue ABTS•^+^ radical cation into a colorless form, which is then measured spectrophotometrically [49]. As shown in Fig. 5a, HA alone, lacking GA functional groups, exhibited negligible radical scavenging activity. In contrast, HA-GA demonstrated a significant increase in antioxidant capacity, reducing more than 90 % of ABTS•^+^, confirming the strong radical scavenging effect of GA moieties. Fig. 5b schematically shows the mechanism of action for GA to quench radicals in ABTS•^+^ solution and transfer the solution color from dark blue to transparent, indicating conversion of GA to the semiquinone form. Interestingly, the addition of KI preserved this high antioxidant capacity (∼92 %), as it was previously reported that KI itself also possesses radical scavenging properties [19]. Here, we also showed that KI alone can have almost an 80 % radical scavenging effect. Similarly, the HA-GA.Gel and HA-GA.μCol groups retained comparably high antioxidant effects, attributed to the combined presence of GA and KI (Fig. 5a). These results confirm that the incorporation of GA and KI into the hydrogel network markedly enhances the radical scavenging ability of the system, which can be beneficial for protecting encapsulated cells from oxidative stress.

To evaluate the protective capacity of the hydrogel formulations against oxidative stress, human dermal fibroblasts (HDFs) were exposed to H_2_O_2_ for 24 h to induce intracellular oxidative damage. Following this treatment, cells were also incubated with various hydrogel precursor solutions, and metabolic activity was assessed using the PrestoBlue assay. In this setup, untreated cells served as the negative control (baseline viability), while H_2_O_2_-treated cells without any further hydrogel treatment represented the positive control (oxidative stress-induced damage). As shown in Fig. 5c, treatment with unmodified HA, gelatin, or collagen alone did not provide any protection, and over 95 % of cells were non-viable, similar to the positive control group. In contrast, cells treated with HA-GA hydrogel showed significantly improved survival, with approximately 23 % viability, highlighting the antioxidant capacity of GA moieties in mitigating oxidative damage. In one of our previous works, we have also shown that the presence of free GA groups in HA gel can reduce oxidative damage and can potentially show anti-inflammatory properties [23]. Notably, when KI was incorporated into the HA-GA formulation, cell survival increased markedly, reaching nearly 60 %, indicating the effect of KI and GA together in scavenging ROS and protecting cells. Similarly, HA-GA.Gel and HA-GA.μCol formulations containing KI also supported over 50 % cell viability, further reinforcing the crucial role of KI in enhancing the antioxidant performance of the hydrogels.

To further evaluate the protective effect of the hydrogels at the cellular level, intracellular ROS levels were assessed using a fluorescence-based ROS detection assay. As shown in Fig. 5d, cells treated with H_2_O_2_ exhibited a strong green fluorescence signal, indicating elevated intracellular ROS levels compared to the untreated negative control group. However, when H_2_O_2_-stressed cells were subsequently treated with HA-GA (no KI), the intracellular ROS level decreased. However, when H_2_O_2_-stressed cells were treated with HA-GA, HA-GA.Gel and HA-GA.μCol formulations containing KI showed a marked reduction in intracellular ROS. This decrease in fluorescence intensity highlights the ability of these hydrogel formulations to reduce oxidative stress within cells. Together, these results emphasize the synergistic antioxidant effect of GA moieties and KI in neutralizing ROS and protecting cells from oxidative damage, which can play a role in immunomodulation.

### 2.6 3D printing and shape-memory

**Fig. 6:**
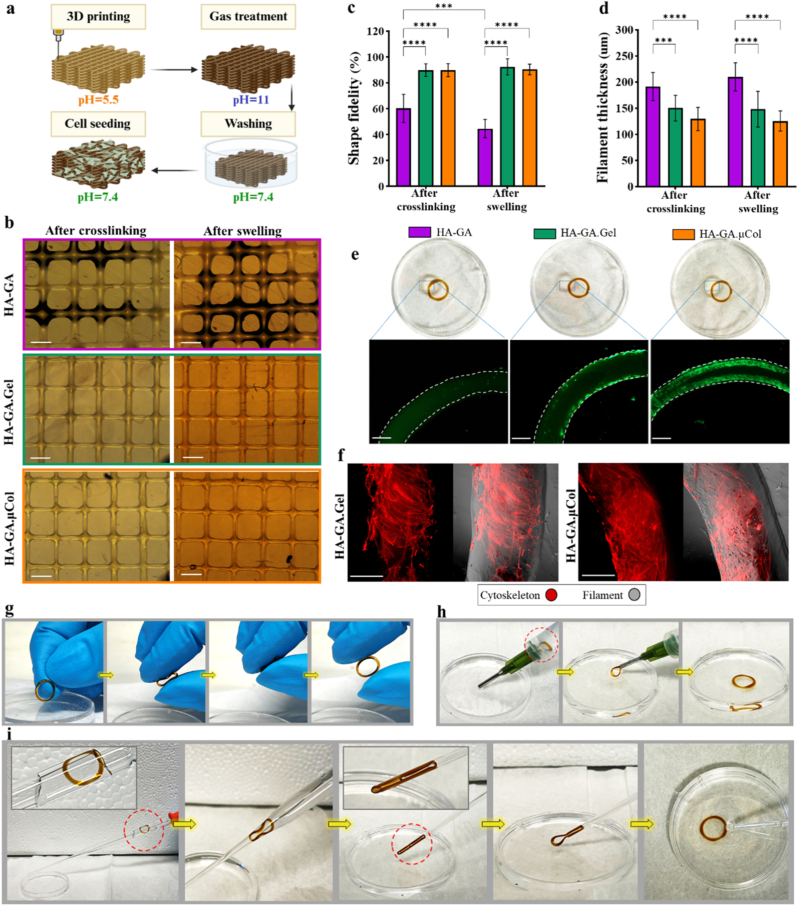
3D printing and shape-memory investigation of hydrogels. (a) Schematic illustration showing the overall workflow: 3D printing of the hydrogel precursor, post-printing ammonia gas treatment for crosslinking, washing with PBS, and subsequent cell loading. (b) Bright field microscopic images of printed constructs made from Gallic modified hyaluronic acid (HA-GA) with different formulations (scale bar: 500 μm). (c–d) Quantitative analysis of shape fidelity and filament thickness of printed structures before and after swelling, using ImageJ software. Statistical analysis is obtained from a two-way ANOVA analysis, significance is represented as: ∗ = p < 0.05, ∗∗ = p < 0.01, ∗∗∗ = p < 0.001, ∗∗∗∗ = p < 0.0001. (e) Fluorescence microscopy images showing live/dead staining of cells loaded on printed hydrogel rings 24 h post-seeding (scale bar: 500 μm). (f) Fluorescent imaging of cells seeded on 3D printed rings after 4 days, stained with phalloidin to visualize the cytoskeleton of cells (scale bar: 100 μm). (g) Digital images showing the shape-memory behavior of the hydrogel ring: complete compression followed by rapid shape recovery after release. (h) Injectability demonstration of printed hydrogel passed through a G14 needle without fragmentation, retaining its original structure post-injection. (i) Visualization of structural compression and recovery using a glass Pasteur pipette, illustrating the folding of the hydrogel inside narrow channels and its immediate shape recovery upon exit.

In the next step, we investigated the potential of the developed hydrogel formulations as inks for 3D printing applications. For 3D printing, we utilized a 32G needle (inner diameter: 108 μm) and an extrusion-based 3D bioprinter, CELLINK BIO-X printer (CELLINK Corporation, Sweden). To achieve printability, we leveraged the two pKa values of the GA moieties in HA-GA. As previously discussed, at pH 5.5, partial oxidation of GA leads to early-stage crosslinking and increased viscosity, without fully forming a solid gel. This transitional state facilitates smooth extrusion through fine needle diameters, while the partially crosslinked state at acidic pH provides sufficient structural integrity for shape retention after printing. Post-printing, the constructs are exposed to ammonia gas to elevate the pH and drive complete oxidative crosslinking. Subsequently, the samples are immersed in PBS to neutralize the pH to 7.4, mimicking physiological conditions (Fig. 6a). Using a 10 mm × 10 mm × 1 mm rectangular prism design with 40 % infill, all hydrogel groups, including HA-GA, HA-GA.Gel, and HA-GA.μCol containing KI were successfully printed (Fig. 6b). Importantly, the printed structures remained intact and retained their form after crosslinking and washing/swelling steps. To quantify the precision of each hydrogel formulation in replicating the intended geometry, shape fidelity was calculated using ImageJ by comparing the printed constructs to the original computational design (Supporting Information, Fig. S11). Fig. 6c demonstrates that among the formulations, HA-GA alone demonstrated the lowest shape fidelity, 60.3 ± 10.8 and 44.6 ± 7.1 % after crosslinking and swelling, respectively. In contrast, HA-GA.Gel and HA-GA.μCol showed significantly improved fidelity (∼90 % both after crosslinking and swelling), which can be attributed to their higher initial viscosities due to the presence of gelatin or μCol. These proteins likely contribute to additional intermolecular interactions with HA and GA at acidic pH, thus enhancing viscosity and structural retention. To note, the shape fidelity calculation inherently captures all deviations that occur immediately after extrusion and before transfer to the ammonia gas chamber, as the structure becomes fixed upon exposure to ammonia and no further significant deformation is possible thereafter. Filament thickness was also assessed post-crosslinking and post-swelling using ImageJ (Fig. 6d). Similar to fidelity results, constructs printed with HA-GA alone exhibited larger filament diameters (191.5 ± 26.7 μm after crosslinking and 210 ± 27.0 μm after swelling), while HA-GA.Gel and HA-GA.μCol maintained more consistent and narrower filaments, ∼150 and ∼125 μm, respectively. Overall, these findings highlight that incorporating gelatin or μCol into the HA-GA matrix significantly improves printability and shape fidelity by enhancing the initial viscosity of hydrogels. Therefore, HA-GA.Gel and HA-GA.μCol are more favorable bioinks for high-resolution printing applications, especially where fine architectural details are critical.

To evaluate the biocompatibility of the printing procedure, a ring with a diameter of 10 mm was printed and similarly treated with ammonia gas, followed by washing in PBS. These 3D printed scaffolds were used to coat with hMSCs in cell culture medium. After incubation, the structures were gently removed, washed with PBS to eliminate loosely attached cells, and stained using a Live/Dead cell viability assay. Fluorescence images in Fig. 6e show that when the ring structure was printed solely from HA-GA, cells were not able to interact with or attach to the ring. However, when gelatin was present in the structure, more cells adhered to the ring, and interestingly, a greater number of cells were able to adhere to the ring printed with HA-GA.μCol. This is due to the higher density and native structure of integrin-binding motifs in collagen, which provide stronger and more specific cues for hMSC adhesion and spreading [50]. Additionally, when the printed hydrogel constructs were maintained under culture conditions for 4 days, a higher number of hMSCs were observed adhering to the surface, and evidence of cell proliferation became apparent, leading to more extensive surface coverage. Fig. 6f shows phalloidin staining of hMSCs cultured on HA-GA.Gel and HA-GA.μCol hydrogels to visualize the cytoskeletal organization which demonstrated both increased cellular coverage and proliferation across the constructs. These results confirmed that the developed 3D printing procedure is safe for cells and preserves the functionality of cell-interactive matrix components.

To demonstrate the shape-memory behavior of the hydrogel, the 3D-printed ring was mechanically compressed with no breakage in the structure (Fig. 6g). Upon release of the applied force, the hydrogel rapidly recovered its original shape, indicating excellent recovery and shape retention. To further assess the injectability of the printed construct, the ring was loaded into a syringe and passed through a G14 needle. As shown in Fig. 6h, the hydrogel could be smoothly injected through the needle without fracturing or losing structural integrity, as it regained its original shape after extrusion through the needle. The shape retention after the injection process is also presented in Supporting Information, Video S1. To visualize the compression and recovery behavior during constrained flow, the same injection procedure was repeated using a glass Pasteur pipette. Fig. 6i shows that the hydrogel maintained its original structure in the wider section of the pipette, but folded into a compressed, nearly 2D configuration within the narrower section. Upon exiting the capillary, the construct immediately returned to its original shape, demonstrating robust shape-memory capability. This process is documented in Supporting Information, Video S2. However, if the hydrogel network were physically fractured during injection, it would not recover its original geometry. This is because GA dimerization forms irreversible covalent bonds, leaving the network without the dynamic bond exchange mechanisms needed for classical shape-healing behavior. Together, these findings confirm that the hydrogel constructs can be printed with high fidelity, subsequently seeded with cells, and delivered non-invasively via injection, retaining both shape and functionality at the target site. However, since the current setup involves printing first, followed by crosslinking through transfer of the samples to an ammonia gas chamber, the achievable structural complexity is limited. If 3D printing could be integrated directly with an ammonia gas environment, more complex geometries could be obtained due to the rapid gelation kinetics and improved shape retention under ammonia exposure. To demonstrate this potential, a hydrogel filament was manually extruded onto pre-fabricated pillars with varying spacing as a proof-of-concept. The filament maintained its shape without collapsing into the gaps between pillars (Supporting Information, Fig. S12). These results indicate that future engineering of a printing system coupled with in situ ammonia mediated crosslinking could enable the fabrication of more diverse and complex structures.

The following are the supplementary data related to this article.

Multimedia component 2 Multimedia component 2

Multimedia component 3 Multimedia component 3

## 3 Conclusions

In this study, we present the development of a novel ECM-mimetic hydrogel that was used as an ink for 3D printing and non-invasive delivery. This ink was designed using GA-modified HA that yielded a single-component-based biomaterial capable of undergoing oxidative crosslinking. The incorporation of KI facilitates radical generation, promotes dimerization of GA groups, and enables covalent conjugation to proteins such as gelatin and collagen, presumably through amino groups. This unique chemistry provides a versatile HA-based backbone that supports the attachment of a wide range of bioactive proteins, allowing for precise modulation of cellular responses. The HA-GA hydrogel takes advantage of pH-responsive behavior governed by the distinct pKa values of the GA moieties. This feature allows the ink to be printed at mildly acidic conditions (ensuring manageable viscosity and fine resolution), followed by complete crosslinking under ammonia gas exposure. The resulting constructs exhibit robust mechanical integrity and long-term stability.

Furthermore, the ability to incorporate protein cues within the hydrogel matrix significantly influences stem cell behavior, including adhesion, elongation, and maintenance of pluripotency markers such as NANOG and OCT3/4. In the presence of growth factors such as TGF-β3, the coated cells could be differentiated into chondrocyte-like cells for potential applications to treat cartilage defects. These features make the system well-suited for engineering biomimetic microenvironments and studying cell–matrix interactions. Importantly, the printed hydrogels demonstrate shape-memory behavior, enabling them to be compressed, injected through fine needles, and recover their original architecture post-injection. Such a characteristic makes this system highly promising for minimally invasive implantation strategies, particularly for applications in cartilage repair, where cartilage defects are clinically treated by transplantation of healthy cartilage tissue to guide tissue regeneration.

Together, these advancements represent a significant step forward in the design of intelligent, bioactive inks for 3D printing, enabling personalized scaffold fabrication, improved delivery, and enhanced therapeutic potential in regenerative medicine.

## 4 Experimental section

### 4.1 Materials

HA (MW 200 kDa) was purchased from LifeCore Biomedical (Chaska, USA). Gelatin from porcine skin, type I, was purchased from Sigma. Collagen microfiber (μCollaFibR™) was purchased from 3DBioFibR. Other chemicals, including 1-ethyl-3-(3-dimethylaminopropyl)-carbodiimide hydrochloride (EDC), 1-hydroxybenzotriazole hydrate (HOBt), and potassium iodide (KI), were purchased from Sigma-Aldrich. Dialysis membranes used for purification were purchased from Spectra Por (Spectra Por-3, MWCO 12–14 kDa).

### 4.2 Synthesis of hyaluronic acid modified with gallic groups (HA-GA)

For this conjugation, 400 mg of HA (1 mmol, 1 eq) was dissolved in 100 mL deionized water in a 250 mL round-bottom flask under constant stirring at room temperature. Subsequently, 135 mg HOBt (1 mmol, 1 eq) was added to the solution. Once HOBt was fully dissolved, 184 mg of gallic hydrazide (GA, 1 mmol, 1 eq) was dissolved in 25 mL of DMSO, which was synthesized following our previous work [23]. This solution was then added dropwise to the HA and HOBt mixture. Thereafter, the pH of the reaction mixture was adjusted to 5.0 using 1M HCl and 1M NaOH, and 57 mg EDC (0.3 mmol, 0.3 eq) was added to the reaction mixture. The pH of the reaction was monitored to ensure it remained stable at pH 5.0 following the addition of EDC. The reaction was carried out overnight under constant stirring at room temperature. Then, the resulting mixture was transferred to a dialysis membrane with a 12–14 kDa molecular weight cutoff and subjected to exhaustive dialysis against 20 L of diluted HCl solution at pH 5 containing 0.5 M NaCl for 48 h. This was followed by dialysis against deionized water without salt for an additional 48 h. The final product (HA-GA) was obtained by lyophilization and stored at −20 °C. The successful grafting of GA onto the HA backbone was confirmed by UV–Vis measurement and ^1^H NMR spectroscopy, which also enabled the determination of the degree of modification to be 20 % by measuring the ratio of the relative peak areas of the aromatic proton peak of GA at 6.89 ppm to the methyl proton peak of the N-acetyl group of HA at 1.95 ppm.

### 4.3 pKa determination

To determine the pKa of GA in HA-GA polymer, we followed a spectrophotometric protocol previously employed in our previous studies [23,51]. Briefly, the working solution was prepared by the addition of HCl and NaOH to deionized water to have the final concentration of 1 and 0.1 mM, respectively. Later, NaCl was added to a final concentration of 0.1 mM, and the solution was kept at room temperature. Then, 10 mg of the HA-GA was added to glass vials and dissolved in 10 mL of the prepared working solution. Due to the photosensitive properties of GA, the solution was covered with aluminum foil. After complete dissolution, the absorbance of the HA-GA solution was measured over a wavelength range of 250–700 nm using a UV–Vis spectrophotometer at different pH levels. The pH of each solution was gradually adjusted in a stepwise manner across a range from pH 3 to 12, and the absorbance was recorded at each pH value. To determine the pKa values, the normalized absorbance was calculated using the equation −log[(Amax − Ai)/Ai] and plotted as a function of pH. The pKa values were identified as the pH at which the fitted curve intersected the horizontal axis on these plots.

### 4.4 Hydrogel preparation

To optimize an ink for 3D printing, three distinct groups of hydrogels were developed: GA-modified HA incorporated with KI (HA-GA group), HA-GA combined with 2 % gelatin and KI (HA-GA.Gel group), and HA-GA combined with 0.5 % micro-collagen fibers and KI (HA-GA.μCol group). The specific compositions of these hydrogels are detailed in Table 1. For preparation, all hydrogel precursors were dissolved in 1X PBS (pH 5), with the solid content of HA-GA set to 4 % and supplemented with 50 mM KI. Once the precursors were fully dissolved, the KI solution was added to the HA-GA mixture and thoroughly blended with the other components using a vortex. The resulting solution was poured into 8 mm cylindrical molds, and crosslinking was initiated by elevating the pH above the pKa of HA-GA. This was achieved by exposing the samples to a 20 % ammonia solution in a closed chamber to get a saturated ammonia gas environment, with a pH of approximately 12–13. Ammonia exposure induced hydrogel formation, as evidenced by a visible color change with a clear transition of gel precursors from a liquid to a gel-like state. After completing the ammonia gas treatment, the hydrogels were washed extensively with 1X PBS (pH 7.4) until their pH equilibrated to 7.4, ensuring biocompatibility for subsequent applications.

**Table 1:** Formulation of hydrogels. The percentages represent the solid content of each polymer component, while KI is expressed by its concentration in the final formulation.

Hydrogels	HA-GA	Gelatin	Collagen	KI
HA-GA	4 %	–	–	50 mM
HA-GA.Gel	4 %	2 %	–	50 mM
HA-GA.μCol	4 %	–	0.5 %	50 mM

### 4.5 Rheological evaluation of hydrogels

To assess the rheological properties of the hydrogels, a TA Instruments TRIOS Discovery HR-2 rheometer was used to measure the storage modulus (G′) and loss modulus (G″) through an amplitude sweep. These values were plotted against strain to illustrate the viscoelastic shear behavior of the hydrogels. Additionally, G′ values at 1 % strain were reported to compare different groups. For sample preparation, 200 μL of each hydrogel formulation was molded into cylindrical shapes (8 mm in diameter) at acidic pH and then cured overnight under ammonia gas. The rheological measurements were performed at this stage, referred to as the ‘as-prepared’ gels. The hydrogels were then washed in PBS for 24 h and remeasured, with this stage referred to as the ‘swelled’ gels.

Gelation time was determined using the same rheometer, with all measurements performed inside a humidified chamber to prevent hydrogel desiccation. After pH adjustment, 500 μL of HA derivative solutions at two different pH levels (acidic and basic) were immediately transferred to the rheometer, and the gap was set to 500 μm. Using a 20 mm parallel plate geometry (strain: 1 %; frequency: 0.5 Hz), the G′ and G″ were recorded over time. Gelation time was defined as the point at which G′ surpassed G''. Additionally, the shear-thinning and injectability behavior of the hydrogels were assessed by measuring viscosity (η∗) as a function of shear strain at both acidic and basic pH.

### 4.6 Swelling and degradation measurements

To assess the stability and swelling kinetics of the hydrogels, HA-GA, HA-GA.Gel, and HA-GA.μCol hydrogels were immersed in a hyaluronidase solution (210 U/mL, approximately 35 times the concentration of the enzyme in human plasma) for the degradation study and in 1X PBS (pH 7.4) for swelling measurement at 37 °C. Briefly, gas-treated hydrogels with 8 mm diameter in a cylindrical shape were transferred to glass vials. The weight of each empty vial, as well as the total weight of the vial containing the hydrogels, was measured. The initial weight of the hydrogels was calculated by subtracting the weight of the empty glass vial from the total recorded weight of the vial with the hydrogels (W_0_). The hydrogels were subsequently immersed in 2 mL of 1X PBS at pH 7.4 for swelling studies and in a hyaluronidase solution to assess their enzymatic degradation rate. The samples were maintained at 37 °C to simulate physiological conditions. To evaluate the swelling and degradation behavior of the hydrogels at predefined time points, the solutions were removed, and the hydrogels were weighed (W_t_). Following this, the PBS buffer and hyaluronidase solution were replaced with fresh solutions, and the samples were kept in the incubator at a steady temperature of 37 °C. The degradation of the hydrogels was determined by calculating the mass change of the hydrogels using equation (1), while the swelling behavior of the hydrogels was assessed by calculating the swelling percentage, as described in equation (2).

(1) $$Mass\;change\;\left(\%\right)=\frac{w_{t}}{w_{0}}$$

(2) $$Swelling\;degree\;\left(\%\right)=\frac{w_{t}-w_{0}}{w_{0}}$$

### 4.7 Radical scavenging evaluation

The free radical scavenging activity of the HA-GA, HA-GA.Gel and HA-GA.μCol hydrogels were assessed by the ABTS (2,2′-azino-bis(3-ethylbenzothiazoline-6-sulfonic acid)) assay. Briefly, the ABTS^+^ solution was prepared by separately dissolving 14.6 mg of potassium persulfate (K_2_S_2_O_8_) in 8 mL deionized water (4.95 mM), which served as the oxidizing agent, and mixing with 8 mL of deionized water containing 31 mg of ABTS. The resulting working solution was stored in the dark and at room temperature for 16 h. Prior to the initiation of the experiment, the pH of the ABTS solution was adjusted to 6.7. Then a concentration of 1 mg of HA-GA, HA-GA.Gel and HA-GA.μCol precursor solution was added into a 12 well-plate containing 0.9 mL ABTS solution per well and incubated in the dark for 15 min. In addition to the mentioned groups, HA, gelatin, micro collagen fibers, and KI were also tested separately. Thereafter, 150 μL of the solution from each well was transferred into a 96-well plate, and the absorbance at 734 nm was measured using a microplate reader. The ABTS scavenging activity was derived using equation (3).

(3) $$ABTS\;scavenging\;activity\;\left(\%\right)=\frac{A_{control}-A_{sample}}{A_{control}}*100$$

Where A_control_ represents the absorbance of the blank ABTS solution without any hydrogel precursor or KI, and A_sample_ represents the absorbance of the ABTS solution in the presence of precursor or KI.

### 4.8 Cell culture

Human mesenchymal stromal cells (hMSCs) and human dermal fibroblasts (HDFs) were separately cultured in T-150 cell culture flasks using Dulbecco's Modified Eagle Medium (DMEM, Gibco) supplemented with 10 % fetal bovine serum (FBS, Gibco) and 1 % antibiotic-antimycotic (AA). Cells from passages 2–6 for hMSCs and 5–10 for HDFs were used for the experiments. Before seeding, the cells were detached from the flasks using TrypLE Select (Gibco), followed by centrifugation and resuspension in a complete medium. After cell counting, the required number of cells was added to each 3D-printed or cast construct.

#### 4.8.1 Live/dead staining of hMSCs

To investigate the impact of each hydrogel composition on cell viability, hMSCs were seeded onto the prepared gels and incubated for 1, 4, and 7 days. Crosslinked HA-GA, HA-GA.Gel, and HA-GA.μCol discs were sterilized by washing in PBS for 24 h, followed by UV exposure for 20 min, and then washed again with complete cell culture medium for 1 h. The samples were then transferred to a 48-well plate, where 30000 cells per well were seeded onto each construct. Subsequently, 400 μL of culture medium was gently added on top, and the plate was placed in a cell culture incubator. The culture medium was replaced every two days. To assess cell viability over time, Live/Dead staining (Viability/Cytotoxicity Kit for Mammalian Cells, ThermoFisher) was performed after 1, 4, and 7 days of incubation. Hydrogels were washed with PBS and then incubated with 300 μL of Live/Dead staining solution (containing 1 μL/mL Calcein AM and 2 μL/mL Ethidium Homodimer in culture medium) for 1 h at 37 °C. After incubation, the hydrogels were washed again with PBS and imaged using a Nikon Eclipse Ts2 fluorescence microscope with a 10 × objective.

#### 4.8.2 Effect of hydrogels on the stemness of hMSCs

To evaluate the effects of hydrogels on the stemness of hMSCs, 50000 cells were seeded onto each hydrogel and covered with the cell culture medium. The samples were incubated at 37 °C for 4 days, after which cells were collected from the gels, lysed using a lysis buffer, and processed for RNA extraction with the RNeasy Plus Mini Kit (Qiagen) following the manufacturer's protocol. Subsequently, cDNA synthesis was performed, and qPCR was conducted to assess fold changes in gene expression levels. For qPCR reactions, cDNA was mixed with TaqMan Fast Advanced Master Mix (Thermo Fisher Scientific), nuclease-free water (Invitrogen), and TaqMan assay primers. The reactions were carried out using a Bio-Rad CFX96 Real-Time PCR machine, following the instructions from the manufacturer. The expression levels of the pluripotency markers NANOG (Hs02387400) and OCT4 (Hs04260367) were analyzed using commercially available TaqMan primers, with ACTB (Hs01060665) serving as an internal control for normalization. Gene expression levels were compared to hMSCs seeded on tissue culture plates (TCPs).

#### 4.8.3 Immunostaining of hMSCs

To examine the effects of hydrogel composition on the cytoskeletal organization (F-actin) of hMSCs, DAPI (6-diamidino-2-phenylindole dihydrochloride) and phalloidin staining (ThermoFisher) were used. Briefly, hMSCs were seeded onto the hydrogels and incubated at 37 °C for 1, 4, and 7 days. After incubation, the hydrogels were washed twice with PBS and fixed with 4 % paraformaldehyde for 30 min at room temperature. Cells were then permeabilized with 0.1 % Triton X-100 (Sigma) for 20 min, followed by PBS washes. To minimize nonspecific background staining, samples were incubated in 0.5 % bovine serum albumin (BSA) in PBS for 1 h. After three additional PBS washes, the cells were stained with DAPI (to label nuclei) and rhodamine phalloidin (to selectively bind F-actin filaments). Finally, the stained samples were visualized under a Leica confocal microscope using a 63 × objective to assess the cytoskeletal organization and morphology of hMSCs. For the quantification of cell coverage area in different groups over time, the ImageJ software was used. In this regard, a threshold-based segmentation approach in ImageJ. The percentage of the scaffold surface covered by cells was calculated relative to the total imaged area.

To evaluate the potential of these scaffolds for cartilage regeneration, hMSCs were seeded onto the printed constructs as described above and cultured in chondrogenic medium containing TGF-β3. The medium was changed every two days with TGF-β3, and after 14 days of incubation, samples were processed for immunostaining to visualize collagen II production. Hydrogels were washed twice with PBS and fixed in 4 % paraformaldehyde for 30 min at room temperature. Cells were then permeabilized with 0.1 % Triton X-100 (Sigma) for 20 min, followed by PBS washes and blocking with 3 % BSA for 1 h. Primary antibody (Anti-Collagen II, ab34712) was added at a 1:200 dilution and incubated overnight at 4 °C. The next day, samples were washed three times with PBS and incubated with a secondary antibody (Goat Anti-Rabbit IgG H&L (Alexa Fluor® 488) (ab150077)) at a 1:300 dilution for 1.5 h. After three final PBS washes, cell nuclei were stained with Hoechst. The stained samples were then visualized using the confocal microscope to assess collagen II expression and confirm chondrogenic differentiation. For quantitative analysis, the fluorescence intensity was normalized to the number of cells using ImageJ software.

#### 4.8.4 Intracellular ROS staining and viability under oxidative stress

To assess the effects of hydrogels on cellular responses under oxidative stress, a 2′,7′-dichlorofluorescein diacetate (DCFH-DA) fluorescence staining assay was conducted. For this assay, 10000 HDFs per well were plated in a 48-well plate and incubated overnight to facilitate cell adhesion. The following day, cells were exposed to 500 μM H_2_O_2_ to induce oxidative stress. Subsequently, hydrogel precursors were introduced into the culture medium at a concentration of 1 mg/mL. A control group without H_2_O_2_ treatment was included as a negative control. The existing medium was replaced with medium supplemented with gel components (1 mg/mL), and cells were incubated for 24 h. After this incubation period, the medium was removed, and the cells were washed twice with PBS. Next, the cells were incubated in a 10 μM DCFH-DA solution for 40 min in the dark at 37 °C. DCFH-DA is a non-fluorescent, cell-permeable dye that undergoes oxidation in the presence of ROS, such as H_2_O_2_, forming the highly fluorescent dichlorofluorescein (DCF). After staining, the DCFH-DA solution was discarded, and cells were washed twice with PBS. Finally, fluorescence microscopy with a 10 × objective was used to capture images and evaluate intracellular ROS levels.

Additionally, a similar experiment was conducted to assess cell viability under oxidative stress. For this, the PrestoBlue metabolic assay was used to evaluate cell viability. Following incubation of the HDFs with the gel precursors (1 mg/mL), they were washed with PBS, and 300 μL of a 10 % PrestoBlue solution (ThermoFisher) was added to each well. The plate was then incubated for 4 h, allowing the reagent to react with metabolically active cells. After incubation, fluorescence intensity was measured using a plate reader with an excitation wavelength of 555 nm and an emission wavelength of 595 nm.

### 4.9 3D printing

For 3D printing, all hydrogel precursors were initially dissolved in an acidic buffer (pH < 5.5). After complete dissolution, KI was added to the solution and thoroughly mixed to prepare the pre-gel formulations of HA-GA, HA-GA.Gel, and HA-GA.μCol. The pH of each solution was then adjusted to 5.5 using NaOH and vortexed to obtain a homogeneous and viscous pre-gel solution. The resulting pre-gel solutions were loaded into sterile plastic printing cartridges (CELLINK, Sweden) and printed using the BIO-X pneumatic extrusion bioprinter (CELLINK Corporation, Sweden) with a 32G needle. Structures with rectilinear infill patterns were designed and printed under optimized conditions. To evaluate printability, a rectangular construct with dimensions of 10 × 10 × 1 mm and 25 % infill density was printed. Additionally, a hollow ring structure was printed for subsequent shape-memory assessments. Following printing, the constructs were placed in a sealed ammonia gas chamber to initiate pH-induced oxidative crosslinking. After complete gelation, the printed structures were transferred to PBS and incubated overnight to remove residual ammonia and equilibrate the pH. Subsequently, the printed rings were utilized for shape-memory experiments, during which they were passed through a glass Pasteur pipette as well as injected through a G14 needle.

To evaluate the biocompatibility and cellular interaction of the printed structures, a pooled population of hMSCs (30000 cells in 500 μL of cell culture medium) was prepared. The printed ring structures were placed in this medium and incubated for 24 h at 37 °C in a humidified cell culture incubator with 5 % CO_2_. After incubation, the structures were gently removed, washed twice with PBS to eliminate loosely attached cells, and stained using a Live/Dead cell viability assay. Fluorescence imaging was performed using a Nikon fluorescence microscope (10 × objective) to assess cell attachment and viability on the hydrogel surfaces. Similarly, after 4 days, the cells were also stained with phalloidin to visualize the cytoskeleton of the cells on the printed ring.

## CRediT authorship contribution statement

**Shima Tavakoli:** Writing – review & editing, Writing – original draft, Methodology, Investigation, Data curation. **Dimitra Pouloutidou:** Writing – original draft, Methodology, Formal analysis, Data curation. **Oommen P. Oommen:** Writing – review & editing, Supervision, Methodology, Formal analysis. **Oommen P. Varghese:** Writing – review & editing, Supervision, Project administration, Methodology, Funding acquisition, Conceptualization.

## Declaration of competing interest

The authors declare that they have no known competing financial interests or personal relationships that could have appeared to influence the work reported in this paper.

## Data Availability

Data will be made available on request.
