# Supplementary material for: Extracellular matrix-mimetic ink for 3D printing and minimally invasive delivery of shape-memory constructs
Source: Mater Today Bio. 2026 Jan 16;37:102818. doi: 10.1016/j.mtbio.2026.102818 (PMC12859500; doi:10.1016/j.mtbio.2026.102818)
Supplement: Multimedia component 1 [file mmc1.pdf]

Supporting Information

Extracellular Matrix-Mimetic Ink for 3D Printing and Minimally Invasive Delivery of Shape-Memory Constructs

Shima Tavakoli[1], Dimitra Pouloutidou[1], Oommen P. Oommen[2], Oommen P. Varghese[1]*

1. Translational Chemical Biology Laboratory, Division of Macromolecular Chemistry, Department of Chemistry-Ångstrom Laboratory, Uppsala University, Uppsala SE75121, Sweden
2. School of Pharmacy and Pharmaceutical Sciences, Cardiff University, United Kingdom CF10 3NB

*Corresponding author

e-mail: oommen.varghese@kemi.uu.se

Schematic representation of hyaluronic acid (HA) modification

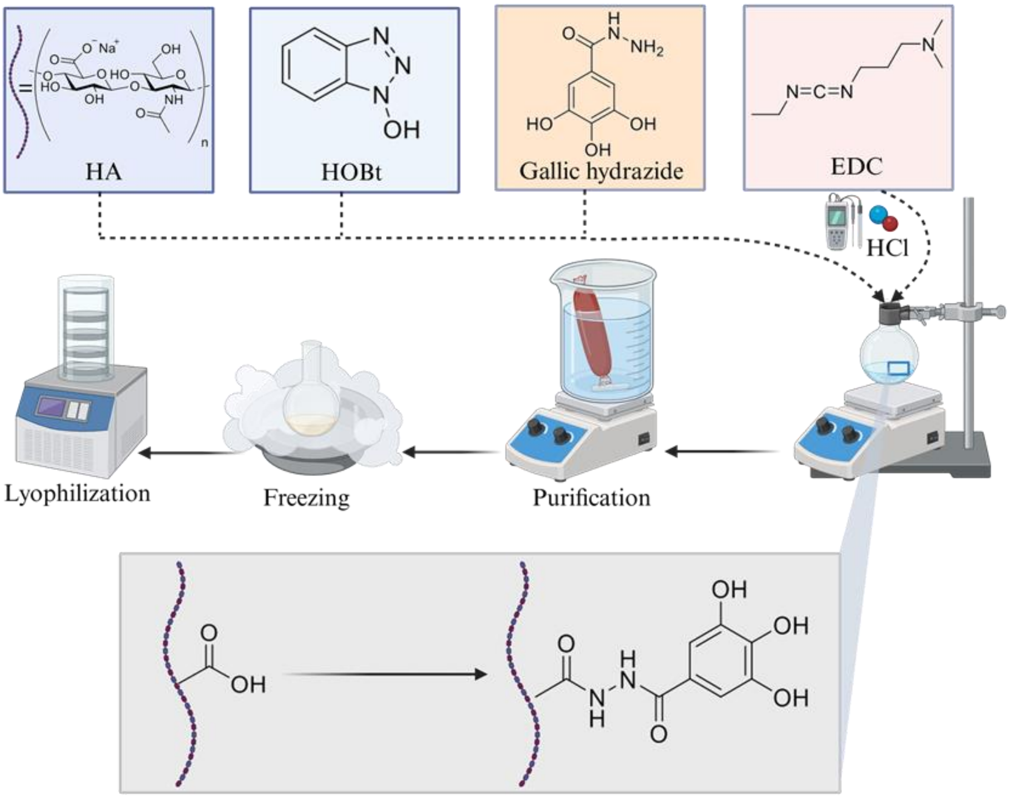

Figure S1. Schematic illustration of hyaluronic acid (HA) modification with gallic hydrazide using carbodiimide chemistry.

Characterization of gallic hydrazide-modified hyaluronic acid (HA-GA)

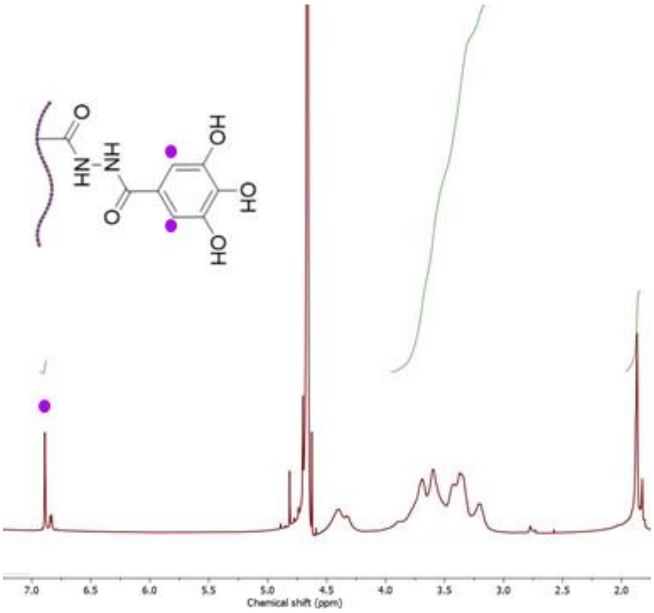

Figure S2. ^1H NMR in D_2O of hyaluronic acid (HA) modified with gallic hydrazide (HA-GA).

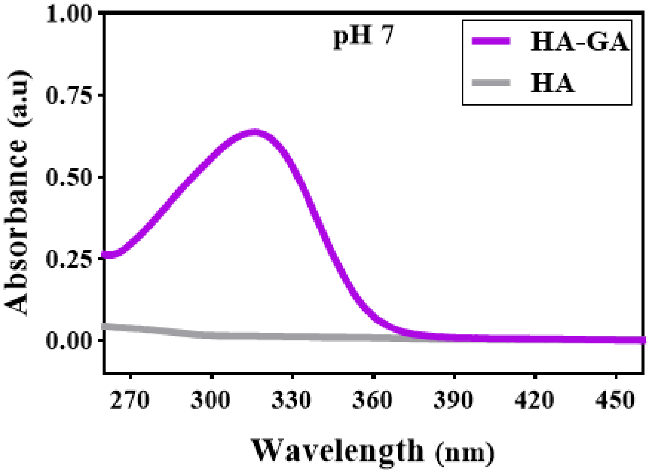

Figure S3. UV-Vis measurement of unmodified hyaluronic acid (HA) and HA modified with gallic hydrazide (HA-GA) at neutral pH.

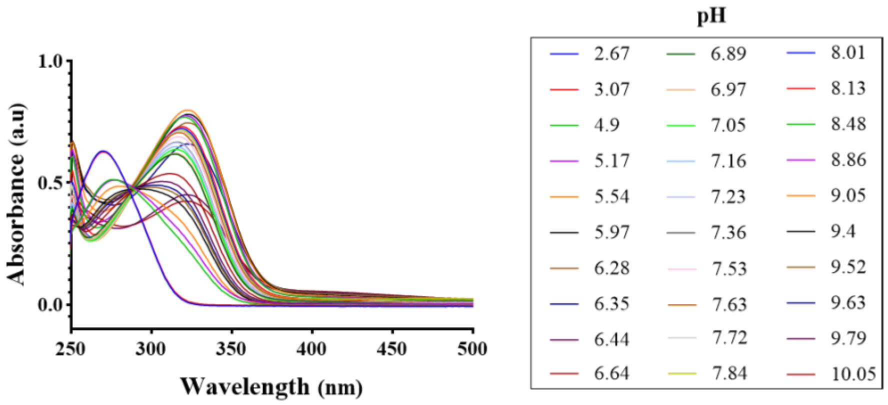

Figure S4. UV-Vis measurement of hyaluronic acid (HA) modified with gallic hydrazide (HA-GA) at different pH.

Crosslinking of HA-GA under ammonia gas in the presence of potassium iodide (KI)

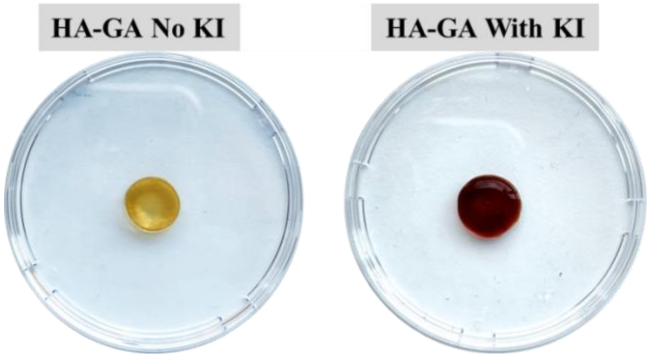

Figure S5. Digital images of HA-GA hydrogels after ammonia gas treatment and subsequent PBS washing, illustrating the role of potassium iodide (KI) in the oxidation of gallic acid groups. The hydrogel without KI appears light yellow, while the presence of KI results in a darker brown color, indicating enhanced radical generation, increased oxidation, and more complete crosslinking of the hydrogel network.

Rheological and physical characterizations of HA-GA

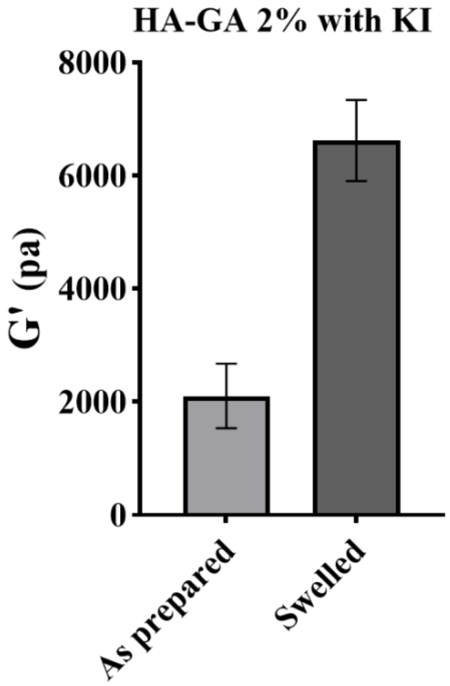

Figure S6. Rheological analysis of gallic hydrazide-modified hyaluronic acid (HA-GA) with 2% solid content and potassium iodide (KI), obtained via amplitude-sweep experiments. Data are shown for hydrogels immediately after ammonia gas crosslinking and after 24 h of swelling in PBS.

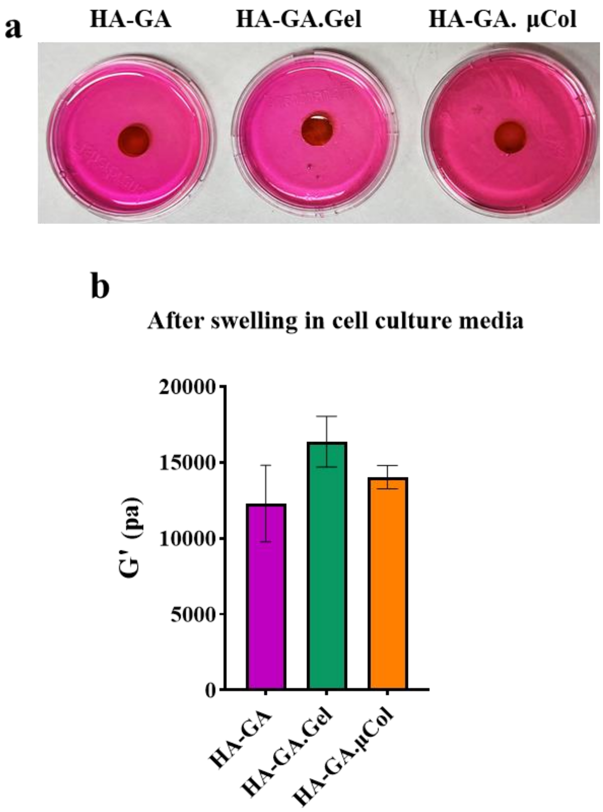

Figure S7. a) Digital representative images of gallic hydrazide-modified hyaluronic acid (HA-GA) alone and also with gelatin (HA-GA.Gel) or collagen (HA-GA.µCol) in the presence of potassium iodide (KI) immersed in cell culture media at 37 ºC, and b) Stiffness of the gels obtained via amplitude-sweep experiments at 37 ºC.

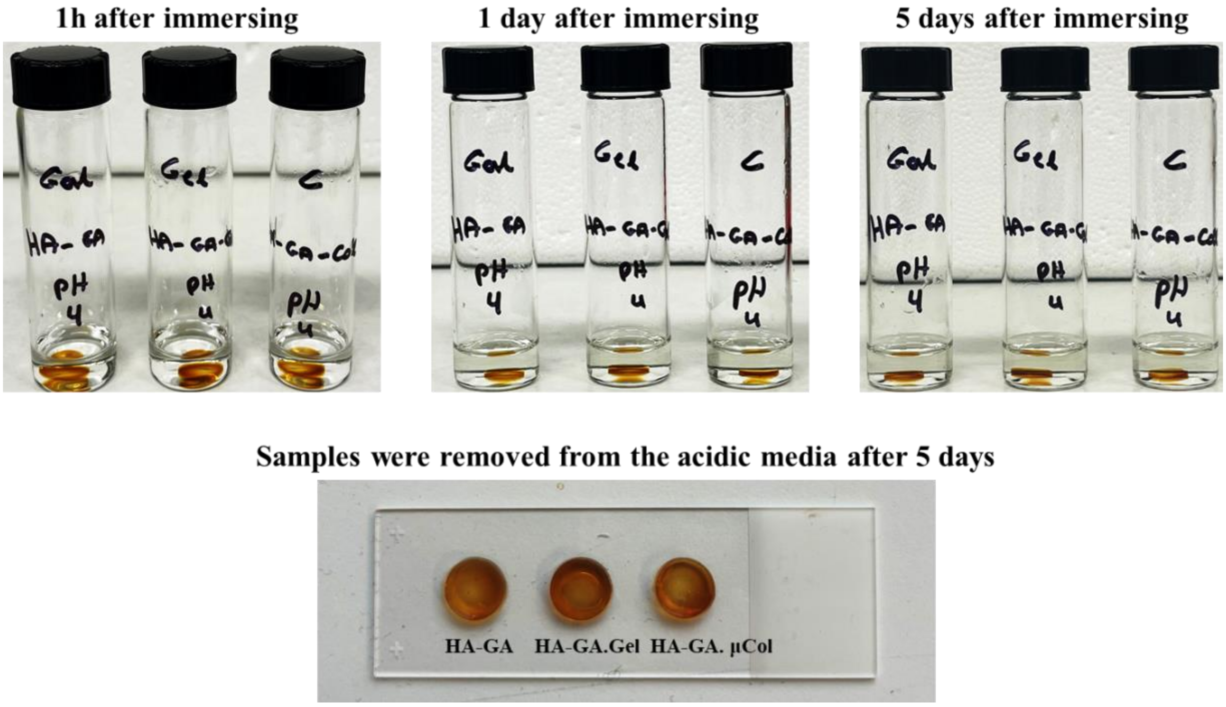

Figure S8. Digital representative images of gallic hydrazide-modified hyaluronic acid (HA-GA) alone and also with gelatin (HA-GA.Gel) or collagen (HA-GA.µCol) in the presence of potassium iodide (KI), immersed in an acidic environment (pH 4) at 37 ºC at different time points.

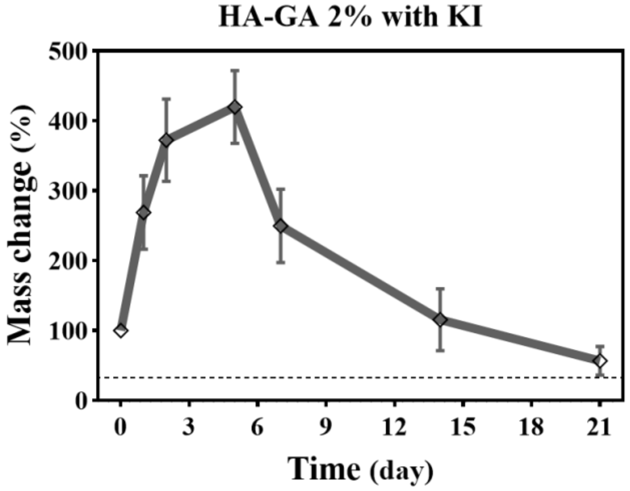

Figure S9. Degradation profile of gallic hydrazide-modified hyaluronic acid (HA-GA) with 2% solid content and potassium iodide (KI), in hyaluronidase solution over 21 days.

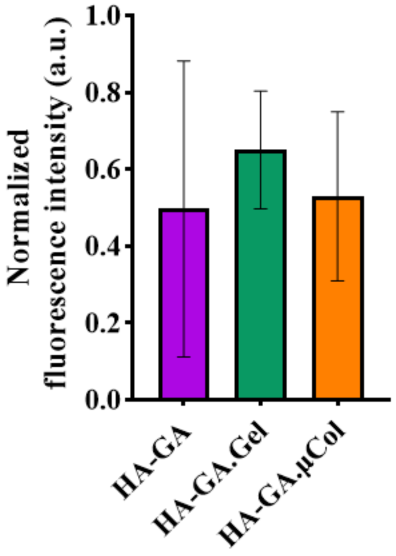

Figure S10. Quantitative normalized fluorescence intensity of Coll II when human mesenchymal stem cells (hMSCs) were cultured on gallic hydrazide-modified hyaluronic acid (HA-GA) alone and also with gelatin (HA-GA.Gel) or collagen (HA-GA.μCol) in the presence of potassium iodide (KI) after 14 days in chondrogenic media.

3D printing evaluation of HA-GA with different formulations

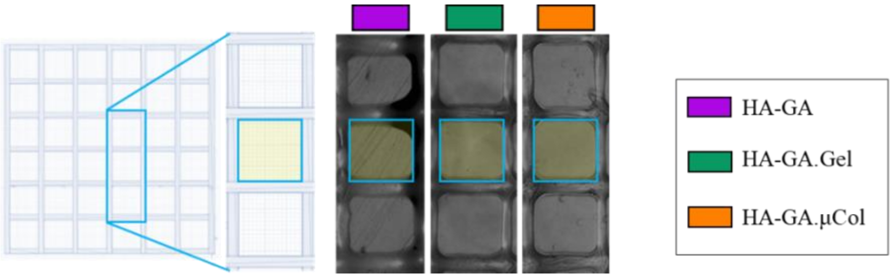

Figure S11. Comparison of 3D-printed structures composed of gallic hydrazide-modified hyaluronic acid (HA-GA), HA-GA with gelatin (HA-GA.Gel), and HA-GA with collagen microfibers (HA-GA.μCol), against the original CAD prism model. Shape fidelity was quantified using ImageJ by measuring key (porosities) structural dimensions and comparing them to the design specifications.

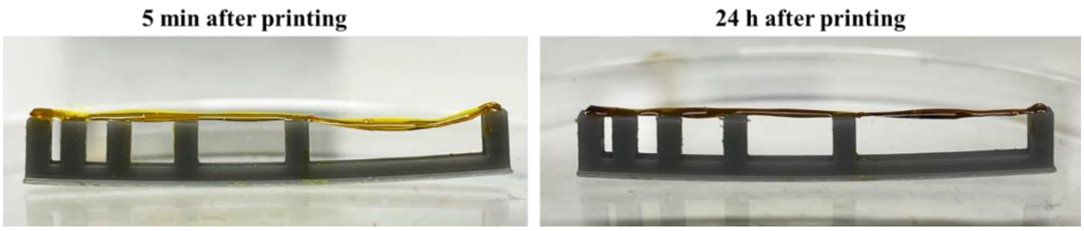

Figure S12. Digital images from the hydrogel string were printed manually on the pillars with different pore distances directly in the ammonia gas chamber.

Video S1. Shape retention of the printed hydrogel ring structure after passing through a G14 needle

Video S2. Shape retention of the printed hydrogel ring structure after passing through a glass Pasteur pipette
